# Gastrointestinal Toxoplasmosis in a Heart Transplant Recipient

**DOI:** 10.1093/ofid/ofag491

**Published:** 2026-09-01

**Authors:** Matthew L Goodwin, Karen P Flores Rosario, Aubrie M Carroll, Avani A Pendse, Madison Swaney, Madeleine R Heldman

**Affiliations:** Division of Infectious Diseases, Department of Medicine, Duke University School of Medicine, Durham, North Carolina, USA; Division of Cardiology, Department of Medicine, Duke University School of Medicine, Durham, North Carolina, USA; Division of Cardiology, Department of Medicine, Duke University School of Medicine, Durham, North Carolina, USA; Department of Pathology, Duke University School of Medicine, Durham, North Carolina, USA; Department of Pathology, Duke University School of Medicine, Durham, North Carolina, USA; Division of Infectious Diseases, Department of Medicine, Duke University School of Medicine, Durham, North Carolina, USA

**Keywords:** heart transplant, prophylaxis, toxoplasmosis

## Abstract

*Toxoplasma gondii* infection poses a significant risk for donor seropositive recipient seronegative heart transplant patients without prophylaxis. Guidelines remain unclear on the optimal duration of prophylaxis in this population. We present a case of disseminated toxoplasmosis presenting as a subacute diarrheal illness leading to fulminant infection with pulmonary involvement in a 38-year-old woman 6 months after stopping primary chemoprophylaxis.

*Toxoplasma gondii* is a recognized opportunistic pathogen that can be fatal in immunocompromised hosts if not promptly recognized and treated [1]. Fever, lymphadenopathy, neurologic symptoms, cytopenias, pneumonia, myocarditis, and uveitis are commonly described presentations of toxoplasmosis in people with advanced HIV and transplant recipients [2]. While gastrointestinal symptoms may also be present, toxoplasmosis is rarely considered to be a primary diarrheal illness [3, 4]. We report a case of toxoplasmosis in a heart transplant recipient who presented with a subacute diarrheal illness and *T. gondii* cysts on gastrointestinal histopathology.

A 38-year-old woman was admitted to the hospital with 1 month of nonbloody diarrhea and an unintentional 20-pound weight loss. Eighteen months prior to presentation, she underwent an orthotopic heart transplant for nonischemic cardiomyopathy. Post-transplant immunosuppression consisted of tacrolimus and rapamycin with a combined goal trough of 10–12 ng/mL and prednisone 5 mg daily; she had no history of rejection. The donor was *T. gondii* seropositive and the recipient was *T. gondii* seronegative prior to transplant (D+/R−). She had completed 1 year of atovaquone prophylaxis for both Pneumocystis and *T. gondii*, which was stopped 6 months prior to presentation. Atovaquone was used given renal dysfunction and hyperkalemia.

On presentation, she was afebrile and oxygen saturation was 98% on room air. She reported intermittent abdominal pain, but no nausea, vomiting, or subjective fevers. She had no sick contacts, denied recent travel, had not consumed any raw or undercooked meat, and had no pets or animal exposures. Laboratory findings showed acute kidney injury on top of chronic kidney disease, with serum creatinine of 3.8 mg/dL from a baseline of 2.0 mg/dL, and mildly elevated transaminases with aspartate aminotransferase (AST) of 71 U/L (reference range 15–41 U/L) and alanine aminotransferase of 42 U/L (reference range 10–39 U/L). Complete blood count noted a mild stable anemia with hemoglobin of 9.6 g/dL, other cell lines were within normal limits. Computed tomography of the abdomen and pelvis without intravenous contrast demonstrated marked mucosal thickening of the colon and stomach, and submucosal fat deposition in the colon consistent with subacute or chronic colitis (Figure 1*A*). Chest radiography was unremarkable. There was no lymphadenopathy on physical exam or imaging. Cytomegalovirus (CMV) was detected in plasma below the limit of quantification (<260 IU/mL) and Epstein-Barr virus was not detected. Rapamycin was discontinued on admission to rule out mTOR inhibitor associated diarrhea.

**Figure 1.**
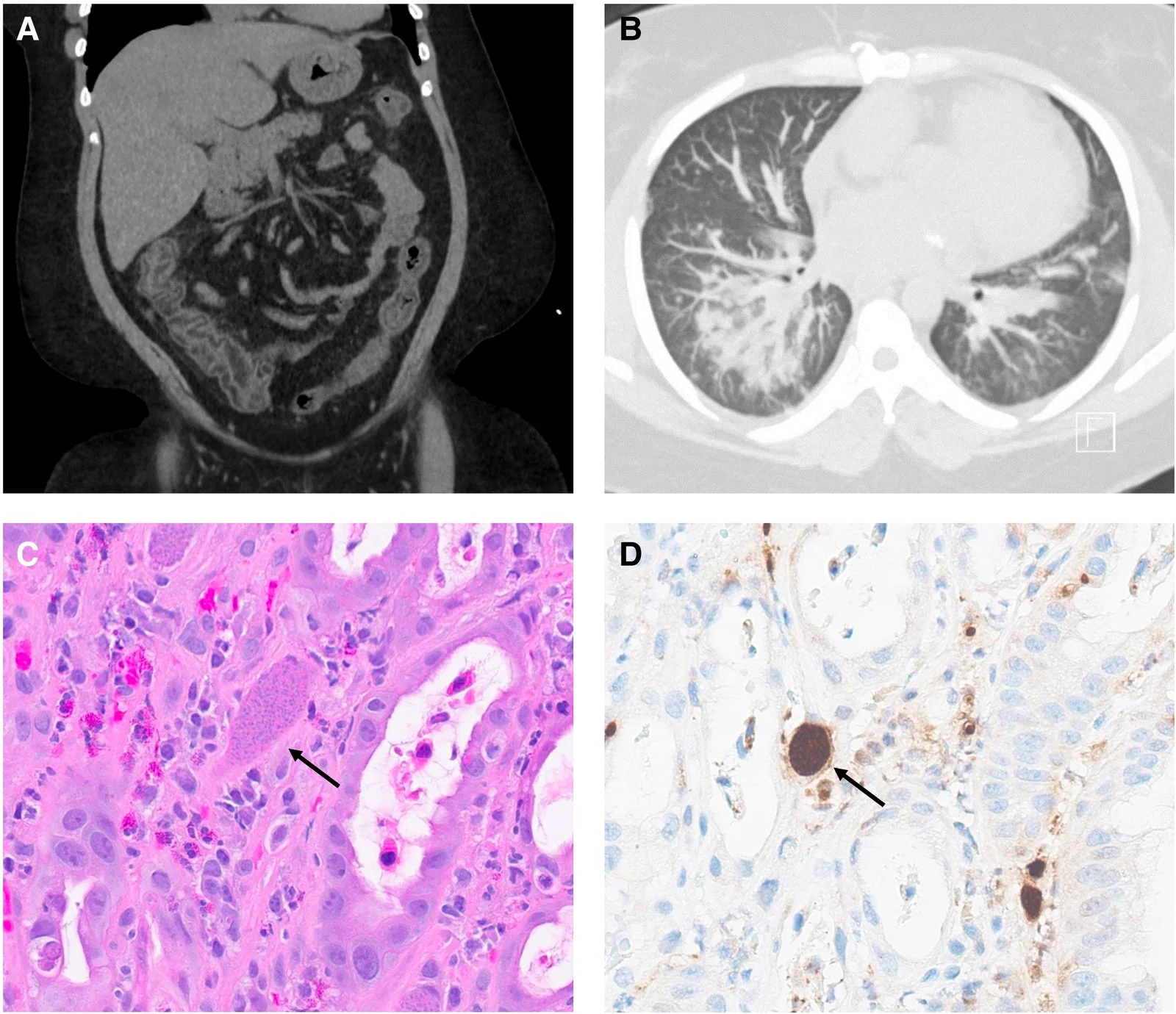
*A*, Computed tomography (CT) of the abdomen in coronal view demonstrating gastric and duodenal mucosal thickening as well as submucosal fat deposition throughout the colon. *B*, CT of the lungs in axial view with bilateral lower lobe predominant nodular and ground glass opacities. *C*, Hematoxylin and eosin-stained slide at 40× magnification showing Toxoplasma tissue cysts (5–100 μm) within gastric lamina propria. The cysts contain the bradyzoite form (1.5–7 μm) of the parasite. There is background acute and chronic inflammation and gastric glands showing reactive changes. *D*, Immunohistochemical stain for *Toxoplasma gondii* at 40× magnification highlights Toxoplasma tissue cysts stained dark brown.

On hospital day 2 she developed intermittent fevers and on hospital day 5 she underwent esophagogastroduodenoscopy (EGD) and flexible sigmoidoscopy, with biopsies obtained from both sites. EGD showed nodular mucosa with multiple nonbleeding ulcers throughout the stomach with normal appearing duodenum. Sigmoidoscopy showed discontinuous areas of ulcerated mucosa throughout the rectum and sigmoid colon and pallor consistent with scarring.

On hospital day 8, before pathology results from endoscopy and sigmoidoscopy returned, her diarrhea worsened and she developed diffuse myalgias, distributive shock, acute renal failure requiring continuous renal replacement therapy, and severe acute respiratory distress syndrome with diffuse alveolar infiltrates (Figure 1*B*) leading to intubation followed shortly by cannulation with veno-veno-arterial extracorporeal membrane oxygenation (ECMO). Transthoracic echocardiogram showed normal left ventricular systolic function without significant valvular pathology and serum creatine kinase was elevated to 2375 U/L, consistent with rhabdomyolysis. The patient was then started on empiric imipenem-cilastatin and micafungin due to concern for septic shock. The fulminant course and high-risk *T. gondii* D+/R− serostatus raised suspicion for disseminated toxoplasmosis, and empiric trimethoprim-sulfamethoxazole (7.5 mg/kg trimethoprim every 12 hours) was also started. On hospital day 9, review of pathology from stomach and colon biopsies demonstrated cystic structures on hematoxylin and eosin (H&E) stain, which stained positive for *T. gondii* by immunohistochemical (IHC) staining (*Toxoplasma gondii* rabbit polyclonal antibody, Cell Marque) (Figure 1*C* and 1*D*). CMV IHC was negative. Whole blood and bronchoalveolar lavage toxoplasma PCRs collected on hospital day 9 resulted at 8 000 000 and 7 900 000 copies/mL, respectively. Imipenem-cilastatin and micafungin were stopped by hospital day 10 following confirmation of toxoplasmosis and negative blood cultures.

On hospital day 10, she remained critically ill with a rising lactate to 13.1 mmol/L and new thrombocytopenia with platelets of 75 × 10^9/L. Serum ferritin was > 1500 ng/mL, triglycerides were 2173 mg/dL, and the erythrocyte sedimentation rate was <1 mm/hour, consistent with secondary hemophagocytic lymphohistiocytosis (HLH). H score for reactive HLH was > 98% [5]. Methylprednisolone 1 g daily was given for 3 days followed by a prednisone taper and anakinra 100 mg IV every 6 hours was started for treatment of HLH [6]. Intravenous immunoglobulin 500 mg/kg was given for 4 days based on experience in treating cytokine syndromes in heart recipients [7]. By hospital day 13, her whole blood lactate improved to 2.0 mmol/L, she was weaned off vasopressors, her arterial ECMO cannula was removed, and she transitioned to veno-venous (VV) ECMO.

The patient underwent tracheostomy on hospital day 15 followed by decannulation from VV ECMO on hospital day 17. Magnetic resonance imaging of the brain and ocular exam did not show any signs of cerebral or ocular toxoplasmosis, respectively. Her course was complicated by persistent renal failure and delirium, with return to baseline mental status by hospital day 39. She completed 6 weeks TMP-SMX (ultimately dosed 7.5 mg/kg q24 hours for intermittent hemodialysis) followed by daily oral TMP-SMX (160–800 mg), with plans for indefinite secondary prophylaxis. At the end of 6 weeks of high-dose TMP-SMX, she continued to have intermittent diarrhea. Repeat stool C-diff testing, multiplex GI pathogen PCR panel were negative. Repeat EGD and flexible sigmoidoscopy showed both gastric and colonic mucosa with features of chronic injury, but pathology showed no signs of *T. gondii* cysts by IHC or on H&E staining. *T. gondii* was not detected on PCR of whole blood. She was discharged from the hospital on day 86 and continued to require hemodialysis.

## DISCUSSION

This case demonstrates a rare presentation of disseminated toxoplasmosis presenting with a subacute gastrointestinal illness that rapidly progressed to multiorgan failure, secondary HLH, and distributive shock requiring mechanical circulatory support. Our patient's mild AST elevation on admission likely reflected early, mild rhabdomyolysis, but no other common manifestations of toxoplasmosis were present at her initial presentation. The marked gastric and duodenal thickening on computed tomography imaging and scattered ulcerations of the stomach, duodenum, and colon have been reported in cases of toxoplasmosis in people with advanced human immunodeficiency virus (HIV) [8], and prompted us look specifically for evidence of toxoplasmosis on gastrointestinal tissue before plasma and BAL PCRs returned. Our patient ultimately made a remarkable recovery with early initiation of TMP-SMX and immunomodulation for secondary HLH, which is a recognized complication of disseminated toxoplasmosis in immunocompromised patients [9].

Among solid organ transplant recipients, *T. gondii* D+R− heart recipients have the highest risk of primary toxoplasmosis, since *T. gondii* tropism for myocytes can lead to transmission through the cardiac allograft. Without prophylaxis, over 50% of *T. gondii* D+R− heart recipients develop *T. gondii* infections in the first several months after transplant [2, 10]. Routine atovaquone or TMP-SMX prophylaxis has reduced the incidence of early toxoplasmosis in *T. gondii* D+/R− heart transplant recipients [11]. Guidelines suggest either 1 year or lifelong prophylaxis in this population and practices vary across institutions [12]. Given our patient's presentation with gastrointestinal symptoms and lack of *T. gondii* microorganisms on post-transplant endomyocardial biopsies, primary infection via foodborne or waterborne transmission followed by dissemination is certainly possible. However, our patient developed severe *T. gondii* infection beyond the first year after transplant without augmentation of immunosuppression or other risk factors for community acquisition, consistent with a late presentation of a transplant-transmitted infection. While this case supports indefinite prophylaxis in *T. gondii* D+R− heart recipients, the absolute risk of toxoplasmosis after stopping prophylaxis 1 year is unknown. Modern epidemiologic studies are necessary to understand the overall risks and benefits of universal, lifelong prophylaxis for all *T. gondii* D+R− heart recipients.

This case highlights *T. gondii* as a gastrointestinal pathogen and serves as a sobering reminder that common infections may have uncommon presentations in immunocompromised patients. Clinicians should consider *T. gondii* as a cause of diarrhea in high-risk immunocompromised patients and evaluate for histopathologic findings of *T. gondii* in appropriate clinical scenarios. While disseminated toxoplasmosis is often fatal, excellent outcomes can occur with early antimicrobial therapy and modulation of secondary inflammatory responses.
